# Exploring User Perspectives of and Ethical Experiences With Teletherapy Apps: Qualitative Analysis of User Reviews

**DOI:** 10.2196/49684

**Published:** 2023-09-22

**Authors:** Eunkyung Jo, Whitney-Jocelyn Kouaho, Stephen M Schueller, Daniel A Epstein

**Affiliations:** 1 Department of Informatics University of California Irvine, CA United States; 2 Department of Psychological Science University of California Irvine, CA United States

**Keywords:** teletherapy, therapy, ethical guidelines, ethics, qualitative research, digital mental health, mobile phone

## Abstract

**Background:**

Teletherapy apps have emerged as a promising alternative to traditional in-person therapy, especially after the COVID-19 pandemic, as they help overcome a range of geographical and emotional barriers to accessing care. However, the rapid proliferation of teletherapy apps has occurred in an environment in which development has outpaced the various regulatory and ethical considerations of this space. Thus, researchers have raised concerns about the ethical implications and potential risks of teletherapy apps given the lack of regulation and oversight. Teletherapy apps have distinct aims to more directly replicate practices of traditional care, as opposed to mental health apps, which primarily provide supplemental support, suggesting a need to examine the ethical considerations of teletherapy apps from the lens of existing ethical guidelines for providing therapy.

**Objective:**

In this study, we examined user reviews of commercial teletherapy apps to understand user perceptions of whether and how ethical principles are followed and incorporated.

**Methods:**

We identified 8 mobile apps that (1) provided teletherapy on 2 dominant mobile app stores (Google Play and Apple App Store) and (2) had received >5000 app reviews on both app stores. We wrote Python scripts (Python Software Foundation) to scrape user reviews from the 8 apps, collecting 3268 user reviews combined across 2 app stores. We used thematic analysis to qualitatively analyze user reviews, developing a codebook drawing from the ethical codes of conduct for psychologists, psychiatrists, and social workers.

**Results:**

The qualitative analysis of user reviews revealed the ethical concerns and opportunities of teletherapy app users. Users frequently perceived unprofessionalism in their teletherapists, mentioning that their therapists did not listen to them, were distracted during therapy sessions, and did not keep their appointments. Users also noted technical glitches and therapist unavailability on teletherapy apps that might affect their ability to provide continuity of care. Users held varied opinions on the affordability of those apps, with some perceiving them as affordable and others not. Users further brought up that the subscription model resulted in unfair pricing and expressed concerns about the lack of cost transparency. Users perceived that these apps could help promote access to care by overcoming geographical and social constraints.

**Conclusions:**

Our study suggests that users perceive commercial teletherapy apps as adhering to many ethical principles pertaining to therapy but falling short in key areas regarding professionalism, continuity of care, cost fairness, and cost transparency. Our findings suggest that, to provide high-quality care, teletherapy apps should prioritize fair compensation for therapists, develop more flexible and transparent payment models, and invest in measures to ensure app stability and therapist availability. Future work is needed to develop standards for teletherapy and improve the quality and accessibility of those services.

## Introduction

### Background

In recent years, the increasingly pervasive use of mobile devices and smartphones has begun to redefine how mental health is managed. Teletherapy apps—stand-alone platforms that specifically conduct synchronous therapy sessions with clients who are not physically colocated with their therapists [1]—have emerged as a potential alternative to traditional in-person therapy, especially after the COVID-19 pandemic [2-4]. Prior work has demonstrated that teletherapy addresses a range of geographical and emotional barriers to accessing care by eliminating the need for individuals living in rural areas to travel [4-7] and by offering anonymity for stigmatized populations [7,8]. In this climate, teletherapy apps have garnered substantial investment and attention as a means to expand access to scale mental health care [9]. Ethical standards guide traditional face-to-face psychotherapy, but their application in digital practice has not been well addressed. In this study, we examined teletherapy apps from the viewpoint of user reviews to understand how ethical principles were followed and incorporated.

The rapid proliferation of teletherapy apps has occurred in an environment in which development has outpaced the various regulatory and ethical considerations of this space. At present, no single regulatory authority oversees the practice of teletherapy apps as professional issues are regulated by state licensing boards, and the Food and Drug Administration regulates apps that provide specific treatments under their regulation of software as medical devices (eg, apps that provide computerized behavioral therapy for psychiatric conditions, such as reSET or Somryst) [10]. Moreover, the Food and Drug Administration relaxed the regulation of mental health apps to facilitate the use of these tools during the COVID-19 pandemic [11,12], which could lead to the widespread adoption of unproven and potentially low-quality mental health apps. Researchers have raised concerns about the ethical implications and potential risks of mental health apps given the lack of regulation and oversight [4,13-15], particularly regarding technical glitches [16-20], business models [19,21,22], and professionalism [16]. However, prior work has focused on general mental health apps more broadly rather than on the ethical aspects of teletherapy apps specifically. As teletherapy apps distinctly aim to more directly replicate practices of traditional care compared with mental health apps, which primarily provide supplemental mental health support, there is a greater need to specifically examine the extent to which teletherapy apps and the care provided through them uphold the ethical standards applied to traditional care.

Thus, we analyzed user reviews of commercial teletherapy apps through the lens of ethical codes of conduct for therapists. All licensed therapists are bound by the ethics code of their profession, such as the Ethical Principles of Psychologists and Code of Conduct [23], Code of Ethics of the National Association of Social Workers [24], and Principles of Medical Ethics [25], and state laws and regulations relevant to their status as licensed professionals. The ethical codes of conduct can serve as good metrics for high-quality care as they provide therapists with general guidelines and expectations for their professional conduct [26]. Prior work has used ethical frameworks to examine existing mental health technology [17,22,27-29], suggesting some important ethical considerations for mental health apps, such as privacy [22,28,30], transparency [17,22], and access to care [22,29].

Although variations exist across the ethical codes of conduct for psychologists [23], social workers [24], and psychiatrists [25], six common themes are central to the ethical practice of therapy:

1. *Professionalism*: ethical guidelines for both psychologists and psychiatrists state that they should uphold professional standards of conduct [23,25].
2. *Continuity of care*: ethical guidelines for both psychologists and social workers suggest that they should make efforts to ensure continuity of care when care is interrupted by different factors. Ethical guidelines require psychologists to “plan for facilitating services in the event that psychological services are interrupted by factors such as...unavailability, or relocation [23].” Guidelines further underline that care should be taken when terminating therapy services. Guidelines for social workers state that they should “avoid abandoning clients who are still in need of services [24].”
3. *Affordability of access*: for example, guidelines for social workers state the following: “Consideration should be given to clients’ ability to pay [24].”
4. *Cost fairness*: guidelines for social workers mention that they should “ensure that the fees are fair, reasonable, and commensurate with the services performed [24].”
5. *Cost transparency*: guidelines for psychologists highlight that the costs involved in therapy should be accurate and transparent, urging them not to “misrepresent their fees [23].”
6. *Access to care*: for example, guidelines for psychiatrists state that they “shall support access to medical care for all people [25].”

Studies have pointed out a lack of research exploring user experiences of publicly available apps for mental health in spite of the critical need to understand users’ real-world experiences to ensure the quality of care provided by mental health apps [22]. Prior work has also pointed out that the evaluation of such apps has primarily focused on professionals, such as therapists, rather than users [16,31-33]. Recent studies have leveraged user reviews of commercial mental health apps as a way to understand user perspectives [16,18-22,34]. User reviews provide a credible source of information for other users to learn about the apps’ benefits and shortcomings [16,34], which could also provide researchers with insights into user perspectives [18,20,21]. Specifically, user reviews can provide concrete examples of the ethical challenges that users face with mental health apps [22]. For example, users have reported that technical glitches interfere with the mental health apps’ ability to provide timely and continuous support during crises [16,22], which can lead to serious emotional consequences for susceptible populations [18]. Users have also expressed concerns about the business models of mental health apps, perceiving those apps as exploitative of population vulnerability [19,21] or nontransparent in the payment process [16,22]. Users further feel that therapists matched through mental health apps are not as professional as in-person therapists [16]. However, there is a lack of understanding of how users perceive teletherapy apps specifically in enacting ethical guidelines, which is crucial for enhancing the quality of the mental health care services provided through these apps.

### Objectives

Examining users’ experiences with teletherapy apps through the lens of ethical codes of conduct can allow us to better understand how users perceive the quality of care that they receive and what considerations are needed to improve the quality of care through such technology. To that end, we examined user reviews of teletherapy apps from app stores through the lens of ethical codes of conduct for therapists. Through our qualitative analysis of the user reviews of teletherapy apps, we unpacked user perspectives and their ethical experiences with teletherapy apps, focusing on whether and how they perceive that such technology enacts the ethical codes of conduct in practice. On the basis of the findings, we discuss the ethical considerations that should be met by teletherapy apps, which will help create standards for how these apps should be designed and how health care policies should be implemented to support individuals’ mental health care.

## Methods

### Data Collection

We searched for potential apps that provide teletherapy services on 2 dominant mobile app stores (Google Play for Android and Apple App Store for iOS) using the search terms “therapy” and “teletherapy.” Teletherapy apps differ from teletherapy that is delivered via other web-based platforms (such as Zoom, Doxy.me, Mend, or SecureVideo) in that, in teletherapy apps, the app company acts as the intermediary of care, whereas on other web-based platforms, the therapist, if in private practice, or health systems remain the intermediaries. For example, on teletherapy apps, the companies provide the terms of service, collect payments, and manage data about patients. Given these differences, we focused on mobile teletherapy apps excluding apps that only function within traditional care facilities (eg, the Anthem Sydney Health app and the Kaiser Ginger app). We carefully read the app descriptions to confirm whether each app provided teletherapy services. We only included apps that had received at least 5000 app reviews on both Google Play and the Apple App Store. The number of reviews is a useful proxy for the quality and impact of apps, allowing us to focus on high-quality apps that are widely used [21]. These criteria yielded 8 apps: BetterHelp, Cerebral, Talkspace, Simple Practice, MDLIVE, Amwell, Doctor On Demand, and Teladoc Health.

We wrote Python scripts (Python Software Foundation) to scrape user reviews from the 8 apps. To extract recent critical user feedback on the apps, we applied the following 3 inclusion criteria for filtering. First, we only included reviews that were written in English. Second, we included reviews of ≥50 characters. Filtering out fake reviews [16] and selecting informative reviews [21] are challenges in using user reviews for research purposes. Excluding shorter reviews helped us address these challenges by improving data integrity and uncovering deeper insights in longer reviews [16]. Third, we limited the reviews to the most recent 500 reviews of each app from Google Play and Apple App Store as of December 6, 2022. Aligned with prior work [16,21], focusing on recent reviews allowed us to concentrate on relevant user experiences of the most up-to-date versions of the apps. Finally, we noticed that some apps (Cerebral, MDLIVE, Amwell, Doctor On Demand, and Teladoc) provide medication management as well as therapy and are mostly focused on providing the former. Therefore, for those apps, we decided to only include reviews that mentioned “therapy” or “therapists.” We read 10% of the collected data and confirmed that the 2 keywords effectively identified reviews that were relevant to teletherapy. As a result, the final data set included 3268 user reviews. The breakdown of the number of reviews analyzed for each app is provided in Multimedia Appendix 1.

### Data Analysis

We used thematic analysis to qualitatively analyze user reviews [35]. In the first phase, the first author (EJ) open coded 1300 user reviews and searched for themes by reviewing and grouping codes into potential themes. We then generated a preliminary codebook with 6 codes (usability, customer support, business model, accessibility, therapist match, and app features) and 17 subcodes. When the entire research team reviewed the preliminary codebook, we noticed that the themes were closely related to ethical principles for mental health professionals, such as billing procedures and access to care. Therefore, in the second phase, we revised the codebook in light of the common themes to the ethical codes of conduct for psychologists, psychiatrists, and social workers. The revised codebook included six parent codes and 13 child codes (the full codebook can be found in Multimedia Appendix 2): (1) professionalism (therapists’ professionalism and therapists’ unprofessionalism), (2) continuity of care (therapist unavailability, service termination, and technical glitch), (3) cost transparency (concerns about cost transparency and appreciation of cost transparency), (4) cost fairness (billing inaccuracy and access-based billing), (5) access to care (overcoming geographical constraints and overcoming social constraints), and (6) affordability of access (affordability and unaffordability). The first (EJ) and second (WK) authors then coded 150 (approximately 5% of the whole data set of 3268) reviews to calculate the interrater reliability, with the κ value for 6 child codes being >0.8 (unaffordability, professionalism, unprofessionalism, overcoming geographical constraints, billing inaccuracy, and cost transparency) and the κ value for 4 child codes being between 0.6 and 0.78 (affordability, overcoming social constraints, service termination, and technical glitch). We had a low agreement for access-based billing (κ=0.3) and therapist unavailability (κ=0.43) in our initial pass. Therefore, we discussed the differences to resolve ambiguities and ensure consistency, which led to more detailed definitions of those codes. Afterward, the 2 authors coded the rest of the user reviews.

### Ethical Considerations

Our research aimed to analyze public app reviews available on commercial app stores. As such, our study did not involve direct interaction with human participants, and all data used for the analysis were publicly available through the app stores. Given that our study solely relied on preexisting and publicly accessible data, it falls under the category of secondary research involving nonidentifiable information [36]. Therefore, we did not seek ethics board review or approval for this study.

## Results

### Overview

The qualitative analysis of app reviews revealed the ethical experiences of teletherapy app users. In this section, we present the findings of the study, focusing on whether and how users’ perspectives and experiences align with the ethical guidelines for mental health professionals. Table 1 summarizes the 6 themes that we identified through the user review analysis. The user review alias indicates which teletherapy app a review was posted for (eg, BetterHelp or Talkspace) on which app store (eg, Google Play or Apple App Store). For example, B-A-1 indicates that the review was for BetterHelp on the Apple App Store.

**Table 1:** Frequency of codes identified across the app reviews and example quotes (N=3268).

Theme and code			n (%)		Example quote
**Professionalism**					
	Therapists’ professionalism	1136 (34.76)		“My therapist constantly checks up on me. I love that you can message them at any time of the day and they usually get back to you fairly fast.” [B-A-162]	
	Therapists’ unprofessionalism	479 (14.66)		“My therapist has canceled her appointment with me 4 times now. This is ridiculous.” [C-G-247]	
**Continuity of care**					
	Technical glitch	540 (16.52)		“The video cuts on and off many times throughout the sessions. Every therapy session had interruptions due to video connection.” [A-G-15]	
	Therapist unavailability	185 (5.66)		“I can’t schedule a meeting with my therapist for MONTHS because there are no available time slots available.” [TD-A-33]	
	Service termination	6 (0.18)		“I was literally responding to the therapist’s question when I was blocked out of sending anything else because I needed to renew the next month. Seriously? That’s how you do your ‘patients’? You cut them out of your app as if it’s a Netflix subscription?” [B-A-55]	
**Affordability of access**					
	Affordability	167 (5.11)		“I am not employed and they have provided me a subscription for low-income people and it has really helped me through this difficult time in my life.” [B-G-208]	
	Unaffordability	151 (4.62)		“Another downside is that [the app] does not accept insurance. So if you have a lower income (like I do), this is not the most affordable option.” [B-A-163]	
**Cost fairness**					
	Access-based billing	159 (4.86)		“The therapists cancel appointments on the same day with no explanation. ...I paid $500+ for one month of service and got literally no help at all.” [T-A-186]	
	Billing inaccuracy	78 (2.39)		“They also continue to charge my credit card despite me canceling the membership multiple times.” [B-A-30]	
**Cost transparency**					
	Concerns about cost transparency	75 (2.29)		“I wish you had told me the price before you took all that information from me. Kind of feel manipulated.” [B-G-205]	
**Access to care**					
	Overcoming geographical constraints	159 (4.86)		“I’d been looking for therapy in my area for years with no luck. It’s a small town with limited options so there was never any opening. Thanks to [this app], I’ve finally had the access I’ve been needing and getting the assistance I need.” [T-G-220]	
	Overcoming social constraints	24 (0.73)		“I have been using this app for a couple of months now and as someone who suffers from social anxiety and finds it uncomfortable just to go out, it has helped me out tremendously.” [TD-A-242]	

### Professionalism

In our study context, professionalism encompasses aspects including therapists’ quality, reliability, and commitment to the therapeutic process. Our findings demonstrate that many users perceived that their therapists met the standards of professionalism as the most frequent code that came up in the reviews was therapists’ professionalism (1136/3268, 34.76%; Table 1). Users often mentioned positive qualities of their therapists, such as being “a good listener” (A-A-457), “non-judgemental” (B-G-472), “understanding” (B-A-208), “compassionate” (S-A-249), and “insightful” (B-A-373).

However, the third most frequent code was therapists’ unprofessionalism (479/3268, 14.66%; Table 1), suggesting that a substantial number of users still perceived that their therapists did not meet the standards of professionalism. Users often perceived that their therapists did not listen to them and their concerns, describing them as “reading from a script” (C-G-130), giving “copied and pasted responses” (T-G-317), and sounding “like robots” (T-A-8). They also criticized the fact that therapists only gave basic and generic responses, describing the quality of therapists’ responses as “fortune cookie grade” (T-A-454). Users further criticized that their therapists were distracted during therapy sessions. Users noticed that their therapists were driving during therapy sessions and were distracted:

It seemed like my therapist had me on speakerphone while she was driving. I definitely knew she was driving because, at one point, she yelled at either another car or pedestrians. She was distracted the entire call.

B-A-235

Users pointed out other unprofessional behaviors from therapists, such as “taking a phone call” (T-A-57) or “falling asleep” (B-A-289) during the sessions. Users developed their own hypotheses regarding the reasons for such unprofessional behaviors from therapists. A user posited that such behaviors resulted from a lack of policies to hold therapists accountable:

[The app] will only guarantee to match you with a therapist. They have no policies in place to ensure the therapist is accountable for actually showing up for an appointment.

B-A-44

A user suspected that therapists had low commitment as the apps were their side jobs:

Every therapist I was paired with made it very obvious that this was their side hustle and they had their own practice that they actually cared about, but not [this app’s] patients.

T-A-489

In addition, users often pointed out that their therapists were not reliable in keeping their appointments and were not available on the timelines that the app promised. Users pointed out that the therapists sometimes did not deliver on the app’s promise of guaranteed response time:

[The app] always says 24-hour guaranteed response window, but the therapists never text back.

T-A-7

They also faced situations in which their therapists repeatedly canceled appointments:

My therapist has canceled her appointment with me 4 times now. This is ridiculous.

C-G-247

A user similarly said the following:

Made two appointments for counseling in a severely distressing time. Both appointments were canceled just minutes before they were to occur.

A-A-12

Users felt that these behaviors were “extremely unprofessional and unethical” (C-G-247) and “absolutely ludicrous” (B-G-103) as it led them to waste their time and money.

### Continuity of Care

Users noted technical glitches, therapist unavailability, and abrupt service termination that affected the teletherapy apps’ ability to provide continuity of care. The app reviews revealed that technical glitches often interfered with users’ ability to engage in continuous care, which was the second most frequent code that came up (540/3268, 16.52%; Table 1). Users frequently mentioned audio or video issues that occurred during their therapy sessions. A user said the following:

The video cuts on and off many times throughout the sessions. Every therapy session had interruptions due to video connection.

A-G-15

Thus, users had to spend time fixing the issues, which shortened the time they were supposed to spend on therapy. A user stated the following:

The constant cutting in and out of the audio takes away much-needed time that could have instead been used to make actual progress in therapy.

S-A-30

A user similarly noted the following:

My therapist nor I could hear each other. I’m really upset about it because two of my appointments were just spent trying to fix this.

S-A-269

Other users mentioned that the therapy sessions ended abruptly because of technical issues. A user said the following:

The calls and live sessions drop so much that it’s incredibly frustrating.

B-G-483

A user similarly noted the following:

We had to disconnect and reconnect multiple times, which causes a disturbance in the flow of conversation.

B-A-229

A user pointed out that such a technical glitch in apps for mental health was irresponsible:

Before you offer an application that’s primary purpose is supporting mental health needs, PLEASE be responsible and ensure it works properly.

T-A-471

User reviews further demonstrated that therapist unavailability compromised the apps’ ability to support continuous care. Users often mentioned that the therapists with whom they had been matched often had limited to no availability in their schedules, which made it challenging for them to engage in weekly therapy as the apps promised. A user said the following:

None of my therapists have been available to meet with me weekly. They are often overbooked or unavailable, and I end up seeing them one or two times per month.

B-A-291

A user similarly noted the following:

I can’t schedule a meeting with my therapist for MONTHS because there are no available time slots available.

TD-A-33

A user also reported that apps failed to provide quick access to care because of therapist unavailability:

I was matched with a therapist who was on time off, which is extremely frustrating because there was a very acute reason for signing up and if I had known that I would have to wait two weeks I’d just go to a normal provider. That’s kind of the whole point of [this app], to be convenient and fast, but it failed at this job massively.

T-G-105

We also found that users experienced a lack of continuity because of the apps’ subscription model. Users mentioned that they lost access to their therapists as soon as their subscription ended without any reminders or warnings. A user said the following:

The app immediately cuts you off from communicating with your therapist once your billing is due. There are no reminders or anything to keep you on your toes they just immediately cut you off.

B-A-17

A user similarly noted the following:

The way they handle payments for subscriptions is manipulative. There is no warning whatsoever that the subscription will expire soon, so you can be prepared to pay to renew it, and once your subscription expires, it will automatically cancel your appointment and lock you out of communicating with your therapist until payment is processed.

B-A-485

A user described how difficult it was when they lost access to their therapist without an opportunity to properly terminate care:

The worst part is if you have an emergency financial hardship, you immediately are unable to inform/speak to your counselor because everything locks if you miss a payment. I really valued the relationship my counselor that I had and she helped me through a lot. The fact that I couldn’t just say goodbye myself was very hard. There should be something against this. I am still battling with anxiety and depression, and this was such a stressful situation not being able to speak to the person who was helping me for months.

B-A-288

A user felt that such practice might be justifiable for generic apps but not for apps that provide mental health care:

I was literally responding to the therapist’s question when I was blocked out of sending anything else because I needed to renew the next month. Seriously? That’s how you do your “patients”? You cut them out of your app as if it’s a Netflix subscription?

B-A-55

### Affordability of Access

Some teletherapy app users perceived that apps provide affordable mental health care by reducing prices compared with traditional in-person therapy. A user mentioned that teletherapy apps provided more affordable options compared with mental health care that she could find where she lived:

I can usually have 3 or 4 sessions in a month for around $260, which you really can’t find that anywhere else in [the city I live].

B-A-336

A user also appreciated that the app provided an affordable option for individuals without insurance:

For the folks like me whose insurance doesn’t cover therapy, the service provided by this app at $65/week is phenomenal. Average [in-person] therapists will cost about $150/week.

B-A-242

Users also valued the fact that the apps provided discounts based on individuals’ financial circumstances even when the apps did not accept insurance. For example, a user described the following:

I am not employed and they have provided me a subscription for low-income people and it has really helped me through this difficult time in my life.

B-G-208

Similarly, a user said the following:

My income looks good, but I’m working through a lot of debt; I e-mailed them and they understood and gave me a bigger discount for a 3-month period.

B-A-119

Users further appreciated that some apps accepted insurance and made care affordable. T-G-220 appreciated that therapy was “covered by my insurance at 100%.” A user similarly mentioned the following:

I put in my insurance member ID, turns out the sessions are FREE.

D-A-434

In contrast, other users perceived that teletherapy apps fell short of their promises of affordability of access as they did not accept insurance. Users thought that the lack of ability to use insurance made teletherapy apps unaffordable, particularly for low-income individuals. A user explained the following:

Another downside is that [the app] does not accept insurance. If you have a lower income (like I do), this is not the most affordable option. This is unfortunate because a lot of people with lower incomes need access to convenient quality mental health care. It is very difficult for those with low income to get therapy because they might not have time due to work or they don’t have a means of transportation to go to face-to-face counseling. If [the app] could make the prices more affordable or at least accept insurance, they would be catering to an untapped market.

B-A-163

A user similarly mentioned the following:

The app is not affordable if you have a minimum wage job. The cheapest plan is over $200 a month. ...I understand this is someone’s career, and they are providing a service. At the same time, the people have severe mental health issues and it’s really unfair that money is the way of us getting help.

T-A-404

Therefore, they desired that apps accept insurance. A user said the following:

I wish that BetterHelp would have a representative for each state to negotiate a way to allow recipients of Medicaid/Medicare to receive a reduced rate. Many of these state-sponsored insurances offer behavioral health coverage. This would open up so much opportunity for some individuals.

B-A-402

They further desired that apps provide flexibility in the payment model rather than sticking to a monthly subscription model with weekly sessions as it would allow them to have more affordable options. A user said the following:

To me, the cost was a HUGE deal breaker. I understand that the therapists need to get paid; however, the fee for unemployed people is $87 per week. It would be nice if it would have given you the option to pay per use and not set up a subscription.

B-G-256

A user similarly noted the following:

They will force you to take weekly sessions, and their so-called discounted price for those weekly sessions is $87 per session. There is no way I could ever afford weekly sessions. At this point, I can only afford monthly or maybe bi-weekly sessions. The fact that they refuse to allow that option is just wrong.

B-G-452

### Cost Fairness

Our findings suggest that users often perceive that teletherapy apps fail to fulfill their commitment to ensuring cost fairness. Users frequently brought up that the subscription model adopted by some apps resulted in them paying fees that were not commensurate with the services that they received. All the apps that primarily provided teletherapy (BetterHelp, Talkspace, and Simple Practice) were based on a monthly recurring billing model that promised weekly therapy sessions. However, because of the aforementioned challenges in ensuring therapists’ professionalism and continuity of care, apps often could not deliver on their promises, which influenced how users perceived the fairness of their cost models. A user pointed out the following:

Three different therapists that either no showed or canceled in the first 2 weeks. So that’s like 2 weeks wasted, $200 wasted.

B-G-257

A user similarly noted the following:

The therapists canceled appointments on the same day with no explanation. ...I paid $500+ for one month of service and got literally no help at all.

T-A-186

Users encountered unfair situations in which they had to pay despite not receiving any care because of therapist unavailability. Users frequently mentioned realizing that therapists had no availability for a few weeks after they had been matched, but the apps often refused to issue refunds citing that users had “access” to mental health care during that time. A user said the following:

I was matched with a counselor that had zero availability for two weeks, yet I was still charged the same.

B-A-133

A user similarly noted the following:

I went to her schedule and there was no availability for the upcoming 3 weeks. Why charge me for a service I don’t even have access to? Now I’m out of $256, with no type of refund in sight.

B-A-397

Users criticized these billing practices, describing them as “unethical” or “predatory*.*” A user stated the following:

I am paying hundreds of dollars for video sessions that are expiring because therapists aren’t taking appointments. ...It is so unethical to take mentally ill people’s money while they’re already struggling and give them nothing in return.

T-A-192

A user also criticized the following:

Predatory payment style. You pay monthly regardless of if they meet with you or not. My therapist had COVID and couldn’t meet with me for multiple weeks. I still got charged for all the time I didn’t get any therapy.

C-G-9

Furthermore, users reported that they were charged although they could not receive care because of the platform’s technical glitches. A user explained the following:

The app nor the website works. I have been charged for 2 months of services and have not gotten the 1st consultation.

B-G-416

A user similarly illustrated the following:

I’ve been charged for a session where the app wouldn’t let me join the session.

T-G-47

### Cost Transparency

The app reviews often manifested users’ concerns about the cost transparency of teletherapy apps, suggesting that the apps were falling short of abiding by the guidelines. Users frequently mentioned feeling frustrated as the cost was not disclosed until they finished the sign-up process, wishing pricing information was clear up front. A user explained the following:

I wish you had told me the price before you took all that information from me. Kind of feel manipulated out of my personal details just to have the help put behind a paywall that isn’t really that affordable.

B-G-205

A user also illustrated the following:

Cost isn’t revealed until the end of the questionnaire. If you can’t afford the therapy, you’ve filled out the whole questionnaire in detail all for nothing, like I just did.

B-G-227

Users further suggested that the apps provided unclear information about which insurances covered them. A user stated the following:

The app is very wishy-washy about how much your insurance will cover upfront. They should tell you what your insurance covers instead of having people guessing.

T-A-339

A user perceived that it was deceiving for apps to advertise that their services were fully covered by insurance when they were not:

They charge you everything at once without telling you exactly what insurance covers. They make it out to be like insurance covers the whole cost.

T-A-400

### Access to Care

Users perceived that teletherapy apps helped promote access to care by allowing them to overcome geographical and social constraints. They appreciated that the apps helped them find therapists when they struggled to find local therapists. Some users mentioned that they were facing difficulties as their local therapists were fully booked:

I had searched for local counselors or therapists for months and was on a number of waiting lists when I finally decided to try [the app].

B-A-3

They appreciated that the apps matched them with therapists promptly:

I tried for a few days calling a local therapist, and they were all booked up weeks in advance. I signed up for [the app], and the next day I was already matched up with a therapist.

B-A-167

Users also valued that the apps helped people living in small towns access care:

I’d been looking for therapy in my area for years with no luck. It’s a small town with limited options so there was never any opening. Thanks to [the app], I’ve finally had the access I’ve been needing and getting the assistance I need.

T-G-220

Users also appreciated that the apps eliminated the need to travel for therapy:

I love this online counseling because the stress to appear in person is reduced tenfold. For me to meet face to face is a stressful 2-hour car ride and a two-hour drive home.

B-G-145

They also liked that teletherapy apps enabled them to continue therapy during the COVID-19 pandemic when in-person therapy was not feasible—“Despite COVID-19 social distancing—when in-person locations became scarce—[the app] made it possible to find a therapist and continue improving my mental health.” (B-A-430)—describing them as a “life saver” (S-A-176) or “lifeline” (S-G-343) during the pandemic.

Some users also felt that teletherapy apps helped them overcome their social anxiety about therapy. These users mentioned their previous struggles with traditional therapy because of their social anxiety:

My intense social anxiety of having to interact with someone in person made me cancel (in-person therapy) frequently. With [the app], I am much less likely to cancel because I can speak to my therapist from the comfort of my own home.

B-A-140

Thus, they valued that teletherapy apps let them engage in therapy without the need to go out:

I have been using this app for a couple of months now and someone who suffers from social anxiety and finding it uncomfortable just to go out it has helped me out tremendously.

TD-A-242

## Discussion

### Principal Findings

Our findings revealed that users’ perceptions of commercial teletherapy apps aligned with some ethical principles of therapy, particularly in terms of promoting access to care and affordability for individuals considered economically disadvantaged. However, users perceived that the apps aligned poorly with other ethical principles such as professionalism, continuity of care, cost fairness, and cost transparency. Some of these problems mirror challenges often experienced in mental health care more generally, such as insurance companies providing ghost networks of therapists (ie, listing therapists who are no longer in the network, are not accepting clients, or have closed their practice) and instances in which therapists display a lack of attentiveness during sessions. However, the unique characteristics of teletherapy apps, such as the remote provision of care, dependence on technology, and existing outside traditional health care facilities, may have exacerbated the challenges in their implementation. Some of these issues could be addressed through straightforward measures such as improving cost transparency. Others require substantially reconfiguring aspects of how teletherapy is conducted, how patients and therapists are matched, and how services are billed in ways that may or may not be possible in current health care systems. In this section, we unpack some ethical implications of our findings and provide some insights for teletherapy apps to ensure the delivery of high-quality care.

### Considerations to Enhance Quality of Care

Our findings suggest that, although teletherapy apps offer the potential for providing affordable care to individuals considered economically disadvantaged, these apps may compromise the quality of care by not adequately compensating therapists at market rates. In our study, we found that many users perceived unprofessionalism among therapists who provided services through teletherapy apps as they often gave basic responses, were distracted by other activities during the therapy sessions, and were not reliable in keeping their appointments. This finding is consistent with prior work indicating that users perceive therapists matched through mental health apps as not as professional and qualified as their in-person therapists as they only provide generic responses and show little interest in their clients [16]. We posit that economic reasons may explain these differences in care. As many teletherapy apps provide cheaper rates compared with in-person therapy settings, therapists tend to receive lower financial incentives on those apps. Although therapists make approximately US $36 per hour on average in the United States [37], therapists on teletherapy apps (eg, BetterHelp and Talkspace) only earn US $14 to US $30 per hour [38]. Therapists in certain states or areas might receive even higher rates, which further reduces the incentive for them to provide high-quality care on these apps. Failure to pay market rates for therapists likely limits their ability to devote sufficient time to meet the expectations that these apps set and compromises the quality of care provided through these apps. Our findings suggest that achieving ethical guidelines of professionalism likely requires a minimum cost to ensure that these therapists are compensated fairly for such work. To ensure quality care, it is critical for teletherapy apps to offer fair compensation. Prior work on apps for depression has highlighted the need to negotiate ways to cover the cost of mental health apps with insurance providers [22]. Our findings similarly pointed to the value of teletherapy apps contracting with insurance providers, Medicaid, and Medicare as it not only would help provide fair compensation for therapists but also might make therapy services available to those who might not otherwise receive care. This might be especially important in areas with mental health care shortages where a sufficient number of providers are not available to meet the demands of those in need. In addition, streamlining operational costs, such as optimizing their technology infrastructure and automating routine administrative tasks, could help balance fair compensation for therapists while ensuring the affordability of teletherapy apps.

Of course, our findings draw from user reviews, which inherently overlook the perspectives of the therapists. Relatively little research has examined therapists’ perspectives on teletherapy apps and the services they provide on them, which is critical for understanding their motivations for participating and perceptions of the quality of care they are able to provide in the setup. Although a few popular press articles have interviewed therapists providing services on these platforms and represented their perspectives, their views may not be representative of the broader views of therapists on these platforms [39,40]. An article noted that the compensation structure for therapists on these platforms often resulted in the need to maintain large caseloads for sufficient pay, which might lead therapists to resort to simplistic responses during their therapy sessions [40]. Furthermore, it was noted that the platform itself may continue to push new clients onto therapists even when their caseloads are full. Another press article highlights how the app companies exert considerable control over therapists’ schedules and engagement with clients and require the use of scripted responses in text chat therapy sessions under certain circumstances [39]. These practices might limit therapist autonomy and lead to practices that might reflect more on the app company and its policies than the care that individual therapists are able to provide. A counterpoint to this view would be that schedules, engagement practices, and scripted responses might increase the quality of care if aligned with best practices, but it is unclear whether this is always the case. Ultimately, more research that centers on the therapist view would be useful, and these articles suggest that app platforms might play a strong role in shaping therapists’ activities on them. Our findings, in conjunction with these insights, underscore the need for future research to delve deeper into the reasons behind therapists’ behaviors on these platforms to develop platform design considerations and potentially even policy that help support care that meets practice standards.

### Developing Flexible Payment Models for Cost Fairness

Our findings also highlight the ethical challenges of using a subscription model for teletherapy apps, particularly regarding cost fairness. In our study, users noted that subscription models of teletherapy apps are potentially problematic, especially when provider availability and activity do not match advertised services included in those subscriptions. All teletherapy apps that were included in our analysis charged their users monthly with the promise of weekly therapy sessions, but they often could not deliver on those promises because of technical glitches and therapists’ unavailability. Although payment models are an important ethical consideration for designing, using, and vetting mental health apps, few studies have paid attention to users’ perceptions of the payment strategies used by those apps [19,21]. Recent work has pointed to the ethical challenges of the freemium payment model of mental health apps, which combines free basic features with advanced subscription services [21]. Such a payment model may exploit vulnerable populations as the complexity of the freemium payment model can result in unexpected charges for users who are experiencing mental health crises. In addition, the limited-term offers associated with the freemium payment model could lead users with ongoing mental health needs to receive incomplete treatments. Aligned with prior work, our findings indicate the ethical challenges of a prevalent payment model used in teletherapy apps, specifically with regard to subscription models.

This subscription model commonly used in teletherapy apps is quite different from that of traditional health care services, which most often follow a fee-for-service model in which users are charged for each service that they receive. A subscription model can be beneficial for the teletherapy app company as it provides a predictable revenue stream based on the number of subscribers. This can help with budgeting and planning for the app’s maintenance and growth to ensure quality care. Subscription models can also encourage users to engage more regularly with the apps as they have already paid for ongoing access to services, which can help improve the effectiveness of the therapy. However, subscription models make it challenging for teletherapy apps to address unexpected circumstances that impede the provision of care. For example, subscription models may not allow for the flexibility needed to address unexpected circumstances that affect therapists’ ability to provide care. If a therapist experiences a personal emergency and cannot keep their appointments, users end up missing out on part of their access to the services that they have paid for. Similarly, if technical issues interrupt a therapy session and a makeup session cannot be scheduled, users also lose partial access to the services that they have paid for.

Prior work has highlighted the need for creating flexible and fair payment models for mental health apps [19,22]. Our study further reinforces the need for teletherapy apps to develop more flexible payment models that account for unexpected circumstances that may affect the provision of care. For example, subscription models could be adjusted to allow for missed appointments or unexpected technical difficulties that interrupt therapy sessions. In addition, a promising way to ensure continuity of care by accounting for unexpected circumstances is value-based care models, or health care delivery models in which providers are paid based on patient health outcomes rather than based on the volume of health care services that they deliver [39-41]. However, these models require contracting with health care systems, insurers, or potentially even states. Innovative projects in this area have been attempted, such as Reno, Nevada, United States, contracting with Talkspace to provide services to its residents [42,43]. Future research on the impact of different payment models (eg, fee-for-service, subscription, freemium, and value-based care) of teletherapy apps on user experience would be beneficial.

### Ensuring App Stability and Therapist Availability

Our findings demonstrate that teletherapy apps provide significant benefits to individuals who face challenges in accessing in-person therapy, including difficulty in finding local therapists, limited ability to travel because of health concerns, and social anxiety. This suggests that teletherapy apps are, to some extent, meeting the expectations set by previous research [13,44] and adhering the ethical guidelines of promoting access to care. However, despite these benefits, our study also revealed several challenges that teletherapy apps face in terms of continuity of care. A significant challenge is the risk of interruptions in care owing to technical glitches. This is consistent with prior work that identified technical issues as a leading cause of negative user experiences with mental health apps [19,20,22,34,45]. Such disruptions can have negative impacts on the mental health of vulnerable populations, who often seek mental health support for urgent reasons [16,18,22]. Our findings suggest a need for developers of teletherapy apps to test their apps more thoroughly than other types of apps to ensure stability and reliability given the sensitive nature of mental health [18]. In addition, prioritizing investment in measures to ensure app stability, high-quality audio and video output, and technical support resources can help mitigate the impact of technical glitches.

Another challenge that we identified was therapist unavailability, as highlighted by prior work on mental health apps [16]. Our findings showed that users frequently realized that their therapists were unavailable for extended periods only after they signed up, which impeded the provision of continuous care. To prevent such situations, teletherapy apps should check users’ schedules and therapist availability *before* charging them. This proactive approach can help ensure that users can receive the care that was promised in their subscriptions. In addition, implementing measures to facilitate prompt matching between therapists and users would be beneficial. It is crucial for teletherapy apps to continuously monitor the user-to-therapist ratio to maintain a proportionate number of available therapists in relation to the number of users who sign up for the service. Maintaining a surplus of available therapists beyond the expected number required to meet the needs of users can also help promote continuity of care. Furthermore, it is important for teletherapy apps to better understand the demographics of their platform to make sure that there are sufficient therapists that meet the needs of their users. Future research is needed to better understand the reasons why teletherapy apps experience a shortage of therapists and how these factors could be addressed to improve the continuity of care.

Our study demonstrated some of the strengths and limitations of teletherapy apps when examined through the lens of ethical guidelines. Future research could be expanded to mental health apps more broadly to consider where these apps conform to or fall short of ethical guidelines as well. Professional organizations and policy makers would benefit from a better understanding of the teletherapy app space as it offers the potential to help expand care to those in need but also has the potential to significantly disrupt practice as we know it. Some of these challenges are easily anticipated, such as expanding considerations for interstate practice with lack of national licensure. Other challenges might be harder to anticipate, such as the growing influence that other advances such as large language models, digital phenotyping, and machine learning might have on these teletherapy apps. Our study used the lens of current ethical guidelines, but it will be important to consider how such guidelines might need to be reconsidered or at least clarify which changes in practice teletherapy apps might generate.

### Limitations

All the selected teletherapy apps were based in the United States. However, how health care systems are designed affects people’s perceptions of and engagement with teletherapy apps as well as traditional in-person mental health care. Therefore, our findings may not be generalizable to other countries with health care systems that are designed differently. In addition, other countries might have different ethical codes for mental health practitioners, which limits our findings to the context of the United States. Further work investigating users’ experiences with teletherapy apps in different countries will also help understand the impact of differences in health care systems and regulation policies on the influence of teletherapy care provided.

Although publicly available user reviews on app stores provided us with insightful data, such a data collection method offered no direct engagement with users. Unlike traditional qualitative methods such as interviews or focus groups, our approach did not allow for further probing of user statements or validation of interpretations of those statements. Furthermore, the analysis of user reviews has inherent biases in the data collected. App reviews are typically provided by individuals who voluntarily decide to share their experiences, which may introduce selection bias as those who choose to write reviews may have more extreme opinions or specific motivations [46]. Specifically in the context of mental health, individuals facing mental health crises might prioritize seeking immediate help and support over writing web-based reviews. As a result, the pool of reviews may not be fully representative of the perspectives of the entire user base. Future work on interviewing a diverse range of teletherapy app users will allow researchers to better capture their ethical experiences with the technology and gain a more comprehensive understanding of user perceptions and experiences. In addition, using app reviews ignores the perspective of the therapists or the app companies, which are important parties to consider when offering guidance on the implementation and regulation of teletherapy apps.

### Conclusions

Our study shed light on users’ perceptions of the services provided in commercial teletherapy apps and aligned these perceptions with ethical guidelines for therapy. Although users appreciated the access to care and affordability of teletherapy apps, they also reported concerns regarding the professionalism of therapists, technical glitches, and therapist unavailability leading to interrupted care and challenges in ensuring cost fairness and transparency under subscription models. Our findings suggest that teletherapy apps should prioritize fair compensation for therapists, develop more flexible payment models, and invest in measures to ensure app stability and therapist availability to provide high-quality care. We hope that this work contributes to ongoing efforts to develop standards for technology-driven mental health care and improve the quality and accessibility of those services.

## Appendix

Multimedia Appendix 1 The number of reviews used for analysis from Android Google Play and the Apple App Store for 8 teletherapy apps.

Multimedia Appendix 2 Themes from ethical guidelines for psychiatrists, psychologists, and social workers.
