# Supplementary material for: Exploring User Perspectives of and Ethical Experiences With Teletherapy Apps: Qualitative Analysis of User Reviews
Source: JMIR Ment Health. 2023 Sep 22;10:e49684. doi: 10.2196/49684 (PMC10559192; doi:10.2196/49684)
Supplement: Multimedia Appendix 1 [file mental_v10i1e49684_app1.pdf]

Multimedia Appendix 1

The number of reviews used for analysis from Android Google Play and the Apple App Store for 8 teletherapy apps

**App name**	**Number of reviews**		
	**Android Google Play**	**Apple App Store**	**Total**
BetterHelp	500	500	1,000
TalkSpace	500	500	1,000
SimplePractice	469	500	969
Cerebral	71	55	126
Teladoc	27	41	68
MDLIVE	27	26	53
AmWell	14	9	23
Doctor on Demand	7	16	23
**Total**			**3,262**
