# Supplementary material for: Exploring User Perspectives of and Ethical Experiences With Teletherapy Apps: Qualitative Analysis of User Reviews
Source: JMIR Ment Health. 2023 Sep 22;10:e49684. doi: 10.2196/49684 (PMC10559192; doi:10.2196/49684)
Supplement: Multimedia Appendix 2 [file mental_v10i1e49684_app2.pdf]

Multimedia Appendix 2

Themes from ethical guidelines for psychiatrists, psychologists, and social workers

Themes	Ethical Codes of Conduct	Type of profession
**Professionalism**	A physician shall uphold the standards of professionalism.	Psychiatrists
	Psychologists uphold professional standards of conduct, [...] and accept appropriate responsibility for their behavior.	Psychologists
**Continuity of care**	Except where precluded by the actions of clients/patients or third-party payors, prior to termination psychologists provide pretermination counseling and suggest alternative service providers as appropriate.	Psychologists
	Psychologists make reasonable efforts to plan for facilitating services in the event that psychological services are interrupted by factors such as the client's/patient's financial limitations, the psychologist's illness, death, unavailability, relocation, or retirement or by the client's/patient's relocation.	Psychologists
	Social workers should make reasonable efforts to ensure continuity of services in the event that services are interrupted by factors such as unavailability, disruptions in electronic communication, relocation, illness, mental or physical ability, or death.	Social workers
	Social workers should take reasonable steps to avoid abandoning clients who are still in need of services. Social workers should withdraw services precipitously only under unusual circumstances, giving careful consideration to all factors in the situation and taking care to minimize possible adverse effects. Social workers should assist in making appropriate arrangements for continuation of services when necessary.	Social workers
**Cost transparency**	Psychologists do not knowingly make public statements (including advertisements) that are false, deceptive, or fraudulent concerning their research, practice, or other work activities or those of persons or organizations with which they are affiliated.	Psychologists
**Cost fairness**	Psychologists and recipients of psychological services reach an agreement specifying compensation and billing arrangements.	Psychologists
	Psychologists take reasonable steps to ensure the accurate reporting of the nature of the service provided [...], the fees, charges, or payments	Psychologists

**Access to care**	Psychologists recognize that fairness and justice entitle all persons to access to and benefit from the contributions of psychology and to equal quality in the processes, procedures, and services being conducted by psychologists.	Psychologists
	A physician shall support access to medical care for all people.	Psychiatrists
	Social workers strive to ensure access to needed information, services, and resources; equality of opportunity; and meaningful participation in decision-making for all people.	Social workers
**Affordability of access**	Psychologists recognize that fairness and justice entitle all persons to access to and benefit from the contributions of psychology and to equal quality in the processes, procedures, and services being conducted by psychologists.	Psychologists
	Consideration should be given to clients’ ability to pay.	Social workers
	A physician shall support access to medical care for all people.	Psychiatrists
